# HIV screening in men and women in Senegal: coverage and associated factors; analysis of the 2017 demographic and health survey

**DOI:** 10.1186/s12879-019-4717-5

**Published:** 2019-12-31

**Authors:** Ndeye Aïssatou Lakhe, Khardiata Diallo Mbaye, Khadime Sylla, Cheikh Tidiane Ndour

**Affiliations:** 1 Clinic of Infectious and Tropical Diseases, Fann National University Hospital, PO: 5035-Fann, Dakar, Sénégal; 2 Parasitology-Mycology Service, Medecine Faculty, Cheikh Anta Diop University, PO: 5005-Fann, Dakar, Sénégal; 3 Division for Fight Against HIV and Sexually Transmitted Infections, Ministry of Health, Dakar, Sénégal

**Keywords:** HIV, Screening, Associated factors, Senegal

## Abstract

**Background:**

Despite the adoption of the provider-initiated HIV testing strategy, the rate of HIV testing is still very low in sub-Saharan Africa. The aim of this study was to assess the factors associated with HIV testing among sexually active women and men in Senegal. Knowledge of HIV status is the gateway to antiretroviral treatment.

**Methods:**

A secondary analysis of the 2017 Senegal Demographic and Health Survey (DHS) was performed, using data on sexually active women aged 15–49 and men aged 15–59. The outcome variable was the proportion of women and men who reported ever being tested for HIV in the last 12 months before the survey. Descriptive, bivariate, and multivariable logistic regression analyses were performed to identify the socio-demographic, HIV-knowledge, media exposure, and behavioral factors associated with HIV testing in Senegal.

**Results:**

The study found that 61.1% (95%CI: 59.2–62.9) of women and 26.2% (95%CI: 24.2–28.3) of men were tested for HIV at the last 12 months. In multivariate analysis, among men the factors independently associated with being tested for HIV were: age groups 20–24 to 40–44 and age group 50–54; a higher level of education; being in the richest household wealth quintile; being married; knowing about the efficacy of HAART during pregnancy; having 2 or more lifetime sex partners and owning a mobile phone. Among women factors independently associated with HIV testing were: being in any age groups versus 15–19; a higher level of education; being in the richest household wealth quintile; being married; knowing about the efficacy of HAART during pregnancy; having any STI in last 12 months; fearing stigma; owning a mobile phone; and having any number of ANC visits, versus none.

**Conclusion:**

Although HIV remains a public health threat, HIV testing’s prevalence is still low in Senegal, making it difficult to interrupt the transmission chain within the community and to reach the UNAIDS goal for 2020 of “90–90-90”. Innovative community-based strategies are needed to address barriers and improve access to HIV testing in Senegal, particularly for men and for the youngest and poorest populations.

## Background

Despite significant progress in recent decades, the HIV epidemic is still a major public health threat worldwide, with an estimated 37 million persons living with HIV in 2017, including 1.8 million children. Africa bears the heaviest burden, with more than 25 million people affected [1]. In Senegal, the AIDS response has achieved notable success since the first case was diagnosed in 1986, with a low and stable prevalence of 0.4% in adults age 15–49 [1], a steady decline in new infections as well as in HIV-related deaths, and a significant increase of 57% in antiretroviral therapy coverage in 2017 [2]. In Senegal, HIV is a female epidemic, with a prevalence of 0.5% in women versus 0.4% in men [3].

The significant advances related to the adoption of early antiretroviral treatment from diagnosis [4] justify the ambitious goal set by the UNAIDS to reach the target of three 90s for 2020—meaning 90% of people with HIV are aware of their HIV status, 90% of those are on antiretroviral therapy, and 90% of those are virally suppressed [5]. UNAIDS predicts that reaching these targets by 2020 would enable the world to end the AIDS epidemic by 2030 [6]. In Senegal, the “Test All, Treat All and Retain in Senegal” strategy, called TATARSEN, was implemented in 2016 and was scaled up at the national level in 2017.

Awareness of HIV testing is key enabling initiation of early treatment and allowing the reduction of HIV-related morbidity and mortality as well as the interruption of the transmission chain. In 2017, however, 25% of all people living with HIV worldwide did not know their HIV status, corresponding at about 9.4 million people living with HIV but unaware of their serological status [1]. The challenge is to increase access to and uptake of HIV testing services (HTS) for those who remain undiagnosed and for those at greatest ongoing risk for HIV infection [6].

In Senegal, Law No. 2010–03 of 9 April 2010 on HIV/AIDS sets the conditions for HIV screening using rapid diagnostic tests (RDTs) even at the health post level and which must be offered anonymously and free of charge. The current strategy is based on voluntary individual testing initiated by patients or care providers [2]. Despite all the progress made, Senegal is still very far from achieving the 2020 target of 90% of people with HIV being aware of their HIV status, with only 69% of person living with HIV diagnosed [2].

In the literature, HIV test uptake was found associated with several individual factors, including age and gender—young people are less likely to seek HIV testing [7–9], while females are more likely to be tested [9–11]. Another factor is person’s sexual orientation and behavior [1, 12–15]. Other identified key factors correlated with HIV testing include area of residence [7, 13], level of education [7, 9, 10], and knowledge of HIV [7, 8, 13, 16, 17]. Also, stigma and discrimination, whether perceived or experienced, has been identified as a real barrier to access to HIV testing services [7, 17, 18].

**Fig. 1:**
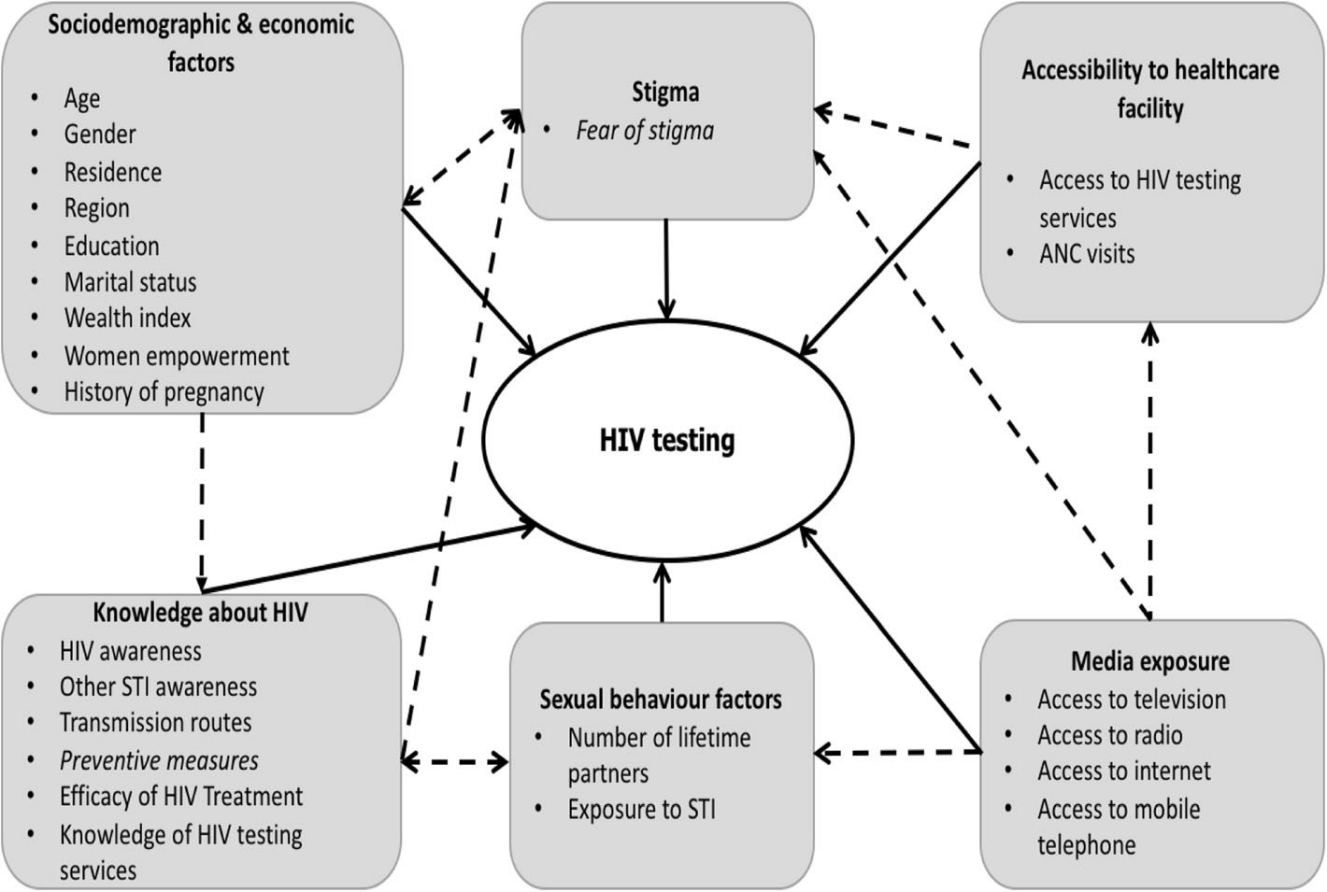
Conceptual framework for HIV testing

To date, no major study of HIV testing has been carried out at the national level in Senegal, and little is known about the correlates of HIV testing. Identifying the factors associated with HIV testing is a critical step in guiding the Senegalese AIDS program to develop policies and strategies, improve progress toward the first UNAIDS 90 indicator and reach the 2020 target. This study assesses factors associated with HIV testing among sexually active women and men in Senegal to better guide screening strategies in the country A conceptual framework has been developed for HIV testing after a literature review and analysis of associated factors. This conceptual framework is illustrated in Fig. 1.

## Methods

### Data sources

The main data source used was the 2017 Senegal Demographic Health Survey (DHS), which is a population-based nationally representative survey in which participants were selected using a two-stage stratified cluster sampling design according to DHS sampling methodology [19]. For the 2017 Senegal DHS, the following methodology was applied [3]: At the first stage, 400 clusters were selected independently with probability proportional to size from the list of enumeration areas (EAs) established during the 2013 General Census of Population and Housing, Agriculture and Breeding (RGPHAE). In the second stage, a sample of 22 households per cluster, both in urban and rural areas, was selected by equal probability systematic sampling. A total of 8800 households (4092 in urban areas and 4708 in middle rural) were selected. In each selected household, a questionnaire was completed to identify women age 15–49, men age 15–59, and children under age 5. Every eligible woman was interviewed with the DHS Woman’s Questionnaire, and in those households selected for the men’s interview, every eligible man was interviewed with the DHS Man’s Questionnaire. These model questionnaires are developed by ICF and are used by several countries. The questionnaires are available in DHS website [20]. After finalizing the collection tools, the survey protocol and questionnaires of the 2017 Senegal Demographic Health Survey (DHS) were sent to the National Ethics Committee (CNERS) for analysis and approval. The CNERS authorized the investigation by the letter N°0035 MSAS/DPRS/CNERS, of April 3, 2017. This survey also obtained the visa of the Committee of Ethics (Institutional Review Board) of ICF. Written informed consent was obtained from all participants prior to the administration of the questionnaires.

**Fig. 2:**
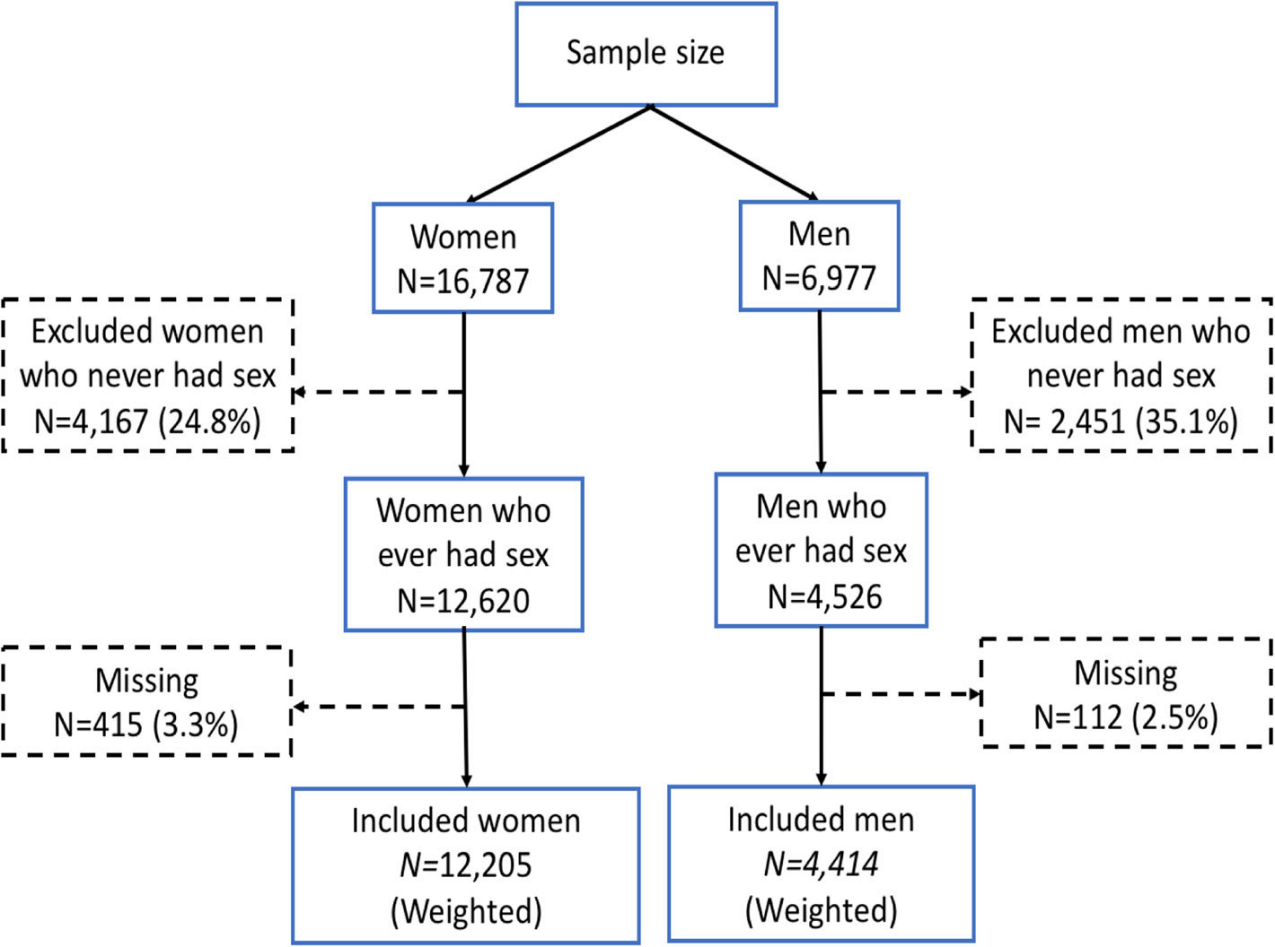
Study population flow diagram

In our study, a secondary analysis of the Senegal 2017 DHS data was performed. Participants from urban and rural areas were selected from all the 14 administrative regions of Senegal. The study focused on sexually active women age 15–49 and men age 15–59. Participants who never had sex were excluded from the datasets, and accounted for 4167 in women and for 2451in men. After considering missing responses, the population size for our study was 12,205 women and 4414 men. The diagram flow of the population of study is represented in Fig. 2.

### Outcome variable

The outcome variable was: ever tested for HIV in the last 12 months. It was measured on the basis of responses to the survey question asked of sexually active men and women: “Have you ever been tested for HIV in the last 12 months?”

### Independent variables

As shown in the conceptual framework, the study considered explanatory variables related to socio-demographic and economic factors, sexual behavior, HIV knowledge, stigma, media exposure, and antenatal care.

Socio-demographic and economic variables included: age (15–24, 25–29, 30–34, 35–39, 40–44, 45–49, 50–54, 55–59); wealth index in quintiles (poorest, poor, middle, rich, richest); and area of residence (urban, rural). Another related variable was residence, with the country’s 14 administrative regions grouped in four geographic zones: West (Dakar, Thiès); North (Saint-Louis, Louga, Matam); Center (Diourbel, Fatick, Kaolack, Kaffrine); South-East (Tambacounda, Kedougou, Ziguinchor, Sedhiou, Kolda). For the analysis, the geographic zone was used rather than the administrative region. Others variables related to socio-demographic factors were: marital status (married, never been in union, divorced, widowed); and level of education (no education, primary, secondary, higher).

Factors related to sexual risk behaviors were: number of lifetime partners (1, 2 and more, don’t know); and history of sexually transmitted diseases (STI) (had any STI in the last 12 months, “*Yes/No*”). Factors concerning knowledge of HIV were: sought knowledge of a place to get HIV test (“*Yes/No*”); knowledge about efficacy of highly active antiretroviral therapy (HAART) during pregnancy (taking drugs to avoid HIV transmission to baby during pregnancy, “*Yes/No*”); and knowledge about HIV and sexually transmitted infections (ever heard of STIs, ever heard of AIDS, knowledge about mother-to-child transmission of HIV). Participants were considered as having a good knowledge of mother-to-child transmission (MTCT) of HIV if they knew the three main transmission routes (pregnancy, delivery, breastfeeding).

To assess perceived HIV-related stigma, the variable was: “people hesitate to take HIV test because of the reaction of other people” (“*Yes/No*”). For media exposure, the variables were: access to the Internet (“*Yes/No*”); and ownership of a mobile phone (“*Yes/No*”).

For women, an additional variable was considered: number of antenatal care (ANC) visits (none, 1 and more). The modality “none” included women who did not have a birth in the last 5 years as well as pregnant women who made no ANC visit.

### Statistical analysis

The analysis was performed using STATA/SE 15.1 software. As stated above in the section on data source, a two-stage sampling design was adopted. To account for the survey’s multi-stage sampling design, all data were weighted to adjust for disproportionate sampling and non-response. For women and men, individual weights were applied.

In the descriptive analysis, variables were presented in terms of the frequency and percentage of data for women and men. Intergroup comparisons were made using Chi2 test. The threshold of significance was set at 5, and 95% confidence intervals (CI) were considered.

To assess the factors associated with HIV testing, two adjusted logistic regressions analyses were performed for women and for men, and adjusted odds ratios (AOR) were calculated with their 95% confidence intervals. To address the complex sampling (multistage sampling, weighting and stratification), the weight, strata, and primary sampling units (PSU) identifier variables were set before using *svy* (STATA survey prefix) command. With the command *svyset*, for variance estimation, Taylor linearization was used. For women, we fit a logistic model of HIV testing with the following 12 independent variables: five-year age groups, zone, educational level, wealth quintile, marital status, knowledge of mother to child transmission of HIV, drugs to avoid HIV transmission to baby during pregnancy, had any STI in last 12 months, lifetime number of sex partners, self-stigma, mobile telephone ownership, and number of ANC visits. For men, a logistic model of HIV testing was fitted with the same independent variables as for women, except the number of ANC visits.

Collinearity was checked and some variables were excluded due to high collinearity with another variable. The variables “residence,” “knowledge of a place to get HIV test”, “television”, “radio” and “use of Internet” were removed from both the models for women and men. For women, the variable “knowledge of a place to get HIV test” was removed because of empty cells, and collinearity was found between “residence” and “wealth class”, and between “televison” and “wealth class”. For men, “knowledge of a place to get HIV test” was removed because of empty cells, and collinearity was found between “residence” and “wealth class”, and “use of Internet” and “educational level”.

## Results

### Background characteristics of the study population

Table 1 summarizes the socio-demographic characteristics of women age 15–49 and men age 15–59 who were sexually active in the 12 months preceding the 2017 Senegal DHS. The greatest proportion of participants in both women and men were aged 30–34, with 19.7% (95%CI: 18.9–20.6) and 16.4% (95%CI: 15.1–17.8) respectively. The majority of women (55.4%; 95%CI: 53.5–57.4) lived in rural areas, while more than a half of men (53.1%; 95%CI: 50.6–55.6) lived in urban ones.

**Table 1:** Distribution of socio-demographic, economic and behaviorial characteristics, knowledge about HIV and others STIs, media exposure and stigma among women aged 15–49 and men aged 15–59 (weighted) [Senegal DHS, 2017]

Variables	Women			Men		
	Percent (%)	95%CI	Number (N)	Percent (%)	95%CI	Number (N)
Socio-demographic and economic characteristics						
Age in 5-year groups						
15–19	7.9	7.2–8.6	964	5.5	4.8–6.4	244
20–24	16.2	15.4–17.0	1974	10.1	9.1–11.1	446
25–29	19.5	18.7–20.3	2377	14.5	13.1–16.1	642
30–34	19.7	18.9–20.6	2407	16.4	15.1–17.8	723
35–39	15.0	14.0–15.9	1825	15.9	14.5–17.4	703
40–44	12.7	12.0–13.5	1555	11.2	10.2–12.4	496
45–49	9.0	8.4–9.7	1103	10.8	9.7–12.0	478
50–54	na		na	8.8	7.8–10.0	389
55–59	na		na	6.6	5.7–7.7	293
Place of residence						
Urban	44.6	42.6–46.5	5437	53.1	50.6–55.6	2344
Rural	55.4	53.5–57.4	6767	46.9	44.4–49.4	2071
Zone						
West	36.8	34.7–38.9	4492	44.2	41.6–46.9	1952
North	16.8	15.6–18.1	2055	15.0	13.3–16.9	661
Center	27.9	26.5–29.4	3,41	19.9	18.5–21.4	879
South-East	18.4	17.3–19.6	2248	20.9	19.3–22.5	923
Educational level						
No education	57.3	55.4–59.1	6989	41.7	38.9–44.5	1,84
Primary	23.3	21.9–24.7	2839	25.1	23.0–27.3	1107
Secondary	16.3	15.2–17.4	1983	24.4	22.4–26.5	1076
Higher	3.2	2.5–4.1	393	8.8	7.0–11.2	391
Marital status						
Never in union	4.9	4.2–5.6	593	30.1	28.0–32.3	1328
Married	88.5	87.4–89.5	10,8	67.7	65.4–69.9	2,99
Widowed	1.2	1.0–1.5	146	0.3	0.2–0.5	12
Divorced	5.5	4.9–6.1	666	1.9	1.4–2.5	84
Never married						
No	95.1	94.4–95.8	11,612	69.9	67.7–72.0	3086
Yes	4.9	4.2–5.6	593	30.1	28.0–32.3	1328
Wealth index						
Poorest	19.5	17.5–21.8	2386	18.1	15.9–20.5	800
Poorer	19.6	17.9–21.5	2397	17.1	15.4–19.0	755
Middle	20.3	18.2–22.4	2473	19.1	16.8–21.7	844
Richer	19.7	17.7–21.9	2407	22.1	19.4–25.1	978
Richest	20.8	18.5–23.4	2542	23.5	20.5–26.8	1038
Knowledge about HIV and STIs						
Ever heard of a sexually transmitted infection (STI)						
No	3.4	3.0–4.0	421	0.8	0.5–1.4	37
Yes	96.6	96.0–97.0	11,784	99.2	98.6–99.5	4378
Ever heard of AIDS						
No	3.7	3.2–4.3	457	1.0	0.6–1.5	43
Yes	96.3	95.7–96.8	11,748	99.0	98.5–99.4	4371
Knowledge about mother-to-child transmission						
No	50.7	48.9–52.5	6189	47.4	45.0–49.7	2091
Yes	49.3	47.5–51.1	6016	52.6	50.3–55.0	2324
Knowledge of a place to get HIV test						
No	19.9	18.4–21.4	2424	37.0	34.4–39.6	1632
Yes	80.1	78.6–81.6	9781	63.0	60.4–65.6	2782
Taking drugs to avoid HIV transmission to baby during pregnancy						
No	52.1	50.0–54.2	6354	45.7	43.2–48.2	2016
Yes	47.9	45.8–50.0	5851	54.3	51.8–56.8	2399
Sexual behavior						
Had any STI in last 12 months						
No	96.7	96.2–97.1	11,8	99.5	99.2–99.6	4392
Yes	3.3	2.9–3.8	405	0.5	0.4–0.8	23
Total lifetime number of sex partners						
1	80.8	79.7–81.8	9859	29.5	27.5–31.6	1302
2+	19.2	18.1–20.2	2339	68.5	66.3–70.5	3023
Don’t know	0.1	0.0–0.1	7	2.0	1.6–2.6	90
Fear of stigma						
People hesitate to take HIV test because of the reaction of other people if positive						
No	22.2	20.7–23.8	2708	22.5	20.7–24.3	992
Yes	77.8	76.2–79.3	9497	77.5	75.7–79.3	3423
Media exposure						
Owns a mobile telephone						
No	29.3	27.6–31.0	3572	6.4	5.6–7.3	282
Yes	70.7	69.0–72.4	8633	93.6	92.7–94.4	4133
Use of Internet						
No	76.9	75.0–78.7	9385	58.0	55.4–60.6	2561
Yes	23.1	21.3–25.0	2819	42.0	39.4–44.6	1854
Numbers of ANC visits						
None	37.3	36.1–38.5	4556	na	na	na
1	2.5	2.2–2.9	306	na	na	na
2–3	23.0	21.9–24.2	2812	na	na	na
4+	37.1	35.9–38.4	4,53	na	na	na
Ever been tested for hiv						
No	38.9	37.1–40.8	4748	73.8	71.7–75.8	3,26
Yes	61.1	59.2–62.9	7457	26.2	24.2–28.3	1155
Received result from last hiv test						
No	41.9	40.1–43.7	5116	5.8	4.5–7.4	67
Yes	58.1	56.3–59.9	7089	94.2	92.6–95.5	1088
Total	100.0		12205	100.0		4415

*na* Not applicable

Nearly six of every ten women (57.3%; 95%CI: 55.4–59.1) had no education versus four of every ten men (41.7%; 95%CI: 38.9–44.5). The proportion with education beyond the secondary level was low both among women (3.2%; 95%CI: 2.5–4.1) and men (8.8%; 95%CI: 7.0–11.2). Nearly nine of every ten women (88.5%; 95%CI: 87.4–89.5) were married compared to two thirds among men (67.7%; 95%CI: 65.4–69.9).

Regarding the household wealth index, 45.6% of men were in the richer and richest quintiles versus 35.2% in the poorer and poorest quintiles. For women, the distribution by wealth quintile was more even, with 40.5% in the richer and richest quintiles versus 39.1% in the poorer and poorest quintiles.

Men had a slightly higher level of knowledge about HIV and sexually transmitted infections (STIs) than women (99.2%; 95%CI: 98.6–99.5 vs 96.6%; CI95%: 96.0–97.0). Surprisingly, awareness of mother-to-child transmission of HIV was also higher among men than women (52.6%; 95%CI: 50.3–55.0 vs 49.3%; 95%CI: 47.5–51.1), even though a higher proportion of women than men (80.1%; 95%CI: 78.6–81.6 vs 63%; 95%CI: 60.4–65.6) knew a place to get an HIV test. Further, more than three quarters of both men (77.5%; 95%CI: 75.7–79.3) and women (77.8%; 95%CI: 76.2–79.3) believed that people hesitate to take HIV test because of the reaction of other people if found HIV-positive.

Media exposure was higher for men, with 93.6% (95%CI: 92.7–94.4) owning a mobile phone versus 70.7% (95%CI: 69.0–72.4) for women, and 42% (95%CI: 39.4–44.6) of men having access to the Internet compared with 23.1% (95%CI: 21.3–25.0) of women.

Regarding the lifetime number of sex partners, nearly height out of ten women (80.8%; 95%CI: 79.7–81.8) had one while more than two thirds of men (68.5%; 95%CI: 66.3–70.5) had two or more.

Among women, 37.1% (95%CI: 35.9–38.4) had four or more ANC visits for their most recent pregnancy while 37.3% (95%CI: 36.1–38.5) had no birth during the past 5 years or did not attend ANC at all. The HIV testing rate in the 12 months before the survey was higher for women than men, at 61.1% (95%CI: 59.2–62.9) versus 26.2% (95%CI: 24.2–28.3).

### Bivariate analysis

Table 2 presents the relationship between uptake of HIV testing and socio-demographic, economic, and behavioral characteristics, knowledge about HIV and other STIs, and media exposure among sexually active women age 15–49 and men age 15–59. For both women and men, uptake of HIV testing was significantly associated with the level of education. Those with a higher level were more likely to get tested. Similarly, regardless of gender, the HIV testing rate increased significantly with the level of household wealth, with the richest respondents being more likely to be tested with 74.2% (95%CI: 70.9–77.4; *p* < 0.001) for women and 40.1% (95%CI: 34.8–45.6; *p* < 0.001) for men. Further, for both men and women, residing in an urban area or in the West zone was significantly associated with HIV testing.

**Table 2:** Association between HIV testing uptake and socio-demographic and economic characteristics’, knowledge about HIV and STI, media exposure ad stigma among women aged 15–49 and men aged 15–59 [Senegal DHS, 2017]

Variables	Ever been tested for HIV within 12 months					
	Women (Yes)			Men (Yes)		
	%	CI	*p*-value	%	CI	*p*-value
Age in 5-year groups						
15–19	34.2	30.5–38.2	< 0.001	17.2	12.7–22.9	0.076
20–24	59.9	56.7–63.1		22.5	18.0–27.8	
25–29	67.6	64.9–70.1		27.5	22.7–32.9	
30–34	68.8	66.1–71.3		27.7	23.2–32.8	
35–39	66.3	63.1–69.4		27.7	23.5–32.3	
40–44	59.1	55.6–62.6		27.2	22.0–33.2	
45–49	50.1	46.0–54.2		26.6	21.7–32.2	
50–54	na	na		30.8	24.2–38.4	
55–59	na	na		20.0	14.6–26.8	
Zone						
West	74.2	71.5–76.7	< 0.001	29.4	25.7–33.5	< 0.01
North	46.4	41.2–51.7		25.7	22.0–29.9	
Center	59.0	55.1–62.7		19.4	16.2–23.1	
South-East	51.6	48.6–54.6		25.9	23.1–29.0	
Place of residence						
Urban	70.6	68.4–72.7	< 0.001	32.5	29.3–35.9	< 0.001
Rural	53.5	50.7–56.2		19.0	16.9–21.2	
Educational level						
No education	53.7	51.4–56.0	< 0.001	13.4	11.5–15.4	< 0.001
Primary	67.3	64.6–69.9		21.7	18.5–25.3	
Secondary	73.8	71.0–76.4		41.5	37.6–45.5	
Higher	84.2	77.8–88.9		56.7	49.0–64.1	
Marital status						
Never in union	55.8	50.4–61.2	0.105	25.9	22.6–29.6	0.296
Married	61.5	59.5–63.6		26.5	24.2–29.0	
Widowed	53.4	44.2–62.3		10.8	2.7–34.3	
Divorced	60.3	54.7–65.6		18.1	11.1–28.1	
Never married						
No	61.4	59.4–63.3	0.051	26.2	24.0–28.7	0.883
Yes	55.8	50.4–61.2		25.9	22.6–29.6	
Wealth index						
Poorest	42.3	38.6–46.2	< 0.001	11.6	9.2–14.6	< 0.001
Poor	52.0	48.4–55.6		19.5	16.4–23.1	
Middle	64.6	61.8–67.3		25.7	22.2–29.6	
Richer	71.3	68.5–74.0		28.7	24.6–33.2	
Richest	74.2	70.9–77.4		40.1	34.8–45.6	
Knowledge about mother-to-child transmission						
No	57.9	55.7–60.0	< 0.001	26.5	24.1–29.2	0.699
Yes	64.4	61.9–66.9		25.8	23.0–28.8	
Taking drugs to avoid HIV transmission to baby during pregnancy						
No	52.7	50.4–55.0	< 0.001	17.3	15.2–19.5	< 0.001
Yes	70.2	68.1–72.3		33.6	30.8–36.6	
Knowledge of a place to get HIV test						
No	0.0		< 0.001	0.0		< 0.001
Yes	76.2	74.7–77.7		41.5	38.7–44.4	
Had any STI in last 12 months						
No	60.7	58.9–62.6	< 0.001	26.2	24.2–28.3	0.561
Yes	71.8	66.3–76.6		21.6	10.4–39.4	
Total lifetime number of sex partners						
1	60.8	58.8–62.9	0.303	19.6	16.9–22.6	< 0.001
2+	62.3	59.8–64.7		28.6	26.2–31.1	
Don’t Know	33.3	5.4–81.5		39.3	29.4–50.1	
People hesitate to take HIV test because of the reaction of other people if positive (Fear of stigma)						
No	39.4	36.6–42.3	< 0.001	18.2	15.2–21.7	< 0.001
Yes	67.3	65.5–69.0		28.5	26.1–30.9	
Owns a mobile telephone						
No	48.4	45.6–51.2	< 0.001	9.9	6.8–14.1	< 0.001
Yes	66.4	64.5–68.1		27.3	25.2–29.4	
Use of internet						
No	56.2	54.1–58.3	< 0.001	15.6	14.0–17.5	< 0.001
Yes	77.4	74.9–79.8		40.7	37.2–44.2	
Numbers of ANC visit						
None	44.3	42.3–46.2	< 0.001			
1+	71.1	69.0–73.2				

HIV testing uptake was significantly associated with age only for women (*p* < 0.001), with adolescents (age 15–19) and young adults (age 20–24) less likely to be tested for HIV than adult women.

Others factors significantly influencing HIV testing were exposure to media, such mobile phone ownership (*p* < 0.001) or use of the Internet (*p* < 0.001) and, for women, the number of ANC visits (*p* < 0.001).

For women, marital status and lifetime number of sex partners were not significantly associated with the HIV testing rate, whereas for men, age, marital status, knowledge of mother-to-child transmission of HIV, and having any STI in the last 12 months were not associated with HIV testing.

### Factors associated with HIV testing

Table 3 summarizes the results of the logistic regression analyses for women and men. In the multivariable analysis for women the following variables were significantly associated with being tested for HIV in the last 12 months: being in any age groups versus 15–19, high level of education; classification in the richest wealth quintile, married, knowledge about the efficacy of HART during pregnancy, any STI in the last 12 months; perceived HIV stigma (people hesitate to take HIV test because of the reaction of other people), ownership of a mobile phone, and number of ANC visits. Women in the North, were less likely to be tested compared with women in the West zone.

**Table 3:** Multivariable logistic model for correlates of HIV testing among sexually active women aged 15–49 and men aged 15–59 in Senegal (DHS 2017)

Variables	Women		Men	
	AOR	95% CI	AOR	95% CI
Age in 5-year groups				
15–19	1.0		1.0	
20–24	2.0***	1.6–2.5	1.3	0.8–2.0
25–29	2.5***	2.0–3.1	1.7*	1.0–2.7
30–34	2.6***	2.0–3.2	1.9*	1.1–3.3
35–39	2.8***	2.2–3.5	2.0*	1.1–3.3
40–44	2.5***	1.9–3.2	1.9*	1.1–3.1
45–49	2.3***	1.7–3.1	1.6	0.9–2.9
50–54	na	na	2.1*	1.1–3.8
55–59	na	na	1.1	0.6–2.1
Zone				
North	1.0		1.0	
West	2.8***	2.2–3.6	0.5***	0.4–0.7
Center	1.9***	1.5–2.4	0.7*	0.5–0.9
South-East	2.1***	1.6–2.7	0.8	0.6–1.1
Educational level				
No education	1.0		1.0	
Primary	1.3***	1.1–1.4	1.7***	1.3–2.2
Secondary	1.8***	1.5–2.1	4.6***	3.5–5.9
Higher	1.9**	1.3–2.9	6.7***	4.6–9.9
Wealth index				
Poorest	1.0		1.0	
Poorer	1.3**	1.1–1.6	1.6**	1.1–2.2
Middle	2.0***	1.6–2.4	2.0***	1.4–2.8
Richer	2.3***	1.8–2.9	2.3***	1.6–3.4
Richest	1.8***	1.4–2.4	2.4***	1.6–3.8
Marital status				
Never been in union	1.0		1.0	
Married	1.6**	1.2–2.1	1.7***	1.3–2.3
Widowed	1.5	0.9–2.7	0.9	0.2–3.6
Divorced	1.4	1.0–2.0	0.8	0.4–1.4
Knowledge about mother-to-child transmission				
No	1.0		1.0	
Yes	1.0	0.9–1.2	0.8	0.6–1.0
Taking drugs to avoid HIV transmission to baby during pregnancy				
No	1.0			
Yes	2.0***	1.7–2.2	1.7***	1.4–2.1
Had any STI in last 12 months				
No	1.0		1.0	
Yes	1.4*	1.1–1.9	0.8	0.3–2.3
Total lifetime number of sex Partners				
1	1.0		1.0	
2+	1.0	0.9–1.2	1.3*	1.0–1.7
Don’t know	1.0	0.1–18.0	2.0*	1.1–3.5
People hesitate to take HIV test because of the reaction of other people				
No	1.0		1.0	
Yes	2.5***	2.2–2.8	1.2	0.9–1.7
Owns a mobile telephone				
No	1.0		1.0	
Yes	1.4***	1.2–1.5	1.8**	1.2–2.7
Number of ANC visits				
0	1.0			
1+	4.2***	3.8–4.7		

*AOR* Adjusted odds ratio *** *p* < 0.001, ** *p* < 0.01, * *p* < 0.05

Among men, the following variables were significantly associated with being tested for HIV in the last 12 months: age groups 20–24 to 40–44 and age group 50–54, a higher level of education, classification in the richest wealth quintile, married, knowledge about the efficacy of HART during pregnancy, and ownership of a mobile phone. Unlike women, men in the North were more likely to be tested compared with men in the West zone.

## Discussion

In Senegal, despite all the efforts made, the rate of HIV testing is still low, but shows a tendency to improve. Our study found that men have a better knowledge of HIV than women have, especially concerning the means of transmission and treatment, and even concerning HIV transmission from mother to child. Despite having lower levels of HIV knowledge, women’s HIV testing rates are much higher than for men, at 62% versus 27%. This seems to be a constant finding across studies [9–11, 21]. It suggests that women have better access to health facilities through several programs devoted to them, such as reproductive health care services. Likely, HIV testing during ANC explains much of the difference between women and men. In fact, ANC visits are opportunities for women to benefit from having an HIV test. Strategies specifically targeting men should be developed in Senegal in order to catch up with women in HIV testing, such as encouraging women during antenatal care to have their partners seek testing.

In our study, adolescents age 15–19 and young adults age 20–24 were less likely to be tested, for both genders. The same observation had been made by other studies. Adolescents are less likely to use HIV testing services [8, 14, 21–24]. Several explanations can be advanced. The level of HIV knowledge among youth in sub-Saharan Africa is very low [8, 17, 25–27] and their use of HIV testing services (HTS) is very poor [21, 25]. This is a problem because teens become sexually active early [21, 25] and are thus more exposed to sexually transmitted infections. In addition, high levels of stigma can be another main cause [14, 17]. HIV-related stigma and discrimination seriously impede efforts to effectively combat the HIV pandemic. Fear of discrimination often prevents people from undergoing HCT or from disclosing their HIV status [28].

To address this gap, we advocate strengthening HIV testing campaigns in secondary schools as well as increasing school-based educational programs. The use of urinary or saliva rapid HIV tests can be promoted as they are associated with HIV testing [29, 30]. Some studies among youth in the African region showed that older youth (20–24 years) had higher odds of HIV testing than younger youth (15–19 years) [8, 21]. Another challenge could be consent for adolescents under age 18. In Senegal, the age of majority is 18, so adolescents under age 18 may not attend HTS. Another explanation of this relationship is that older age confers more economic and social power, which is why among both women and men older people are more likely to be screened for HIV.

Another factor is that after age 25 most women and men are married. With pregnancy, women gain access to antenatal care services and have more opportunities to be tested. Senegal adopted universal HIV testing during pregnancy as recommended by WHO [6]. In line, as expected, our study found that women with at least one ANC visit had four times the odds of having an HIV test compared with those who had no ANC visit or did not have a birth in the last 5 years.

Women living in the West zone are more likely to be tested than women in the other zones of Senegal. The West zone includes the regions of Dakar and Thiès—the capital city and its closest region—so many health services are concentrated there, and people also have better access to the media and greater economic power. This leads to increased opportunities for HIV testing. Also, these areas are predominantly urban. However, the results for men are in the opposite direction than women. Men living in North and South-East zones are more likely to be tested. The reason behind this discrepancy has to be examined more closely. However, studies show that, regardless of gender, people living in urban areas are more likely to be tested for HIV compared with their counterparts in rural areas [7, 13, 24, 31]. HIV testing strategies must target HIV hot-spot regions, specifically in the South-East zone.

Our study highlights how difficult it is for health promotion programs to reach the poorest sections of the population. Even though HIV testing in Senegal is free, testing rates increase with educational level and wealth for both men and women. These findings are consistent with previous reports [7, 9, 10, 12, 13, 24, 31, 32]. On average, the odds of having HIV testing are 6.3 times higher among men and 2.8 times higher among women with a high level of education compared with men and women with no education. Higher educational attainment is associated with more comprehensive knowledge of HIV and its prevention [32, 33]. Women deciding alone about their own health care are more likely to be tested for HIV compared with women for whom someone else is involved in decisions on their care, whether husbands/partners or other family members.

In agreement with studies in Ethiopia and Rwanda [7, 14], we found that media exposure increases the uptake of HIV testing. For many years now in Senegal, messages extolling the benefits of early detection and treatment have been distilled through the media, particularly on television and radio. However, these campaigns have shown their limits, while other strategies exist that can reach audiences directly. In our study both men and women show greater odds of HIV testing if they own a mobile phone. Since it appears that, regardless of gender, possession of a mobile phone is a predictor of HIV testing, strategies to reach men and women through their mobile phones should be developed and implemented.

STI status is another important determinant for HIV testing [10, 24, 34]. Our study did not allow us to draw this conclusion for women, however. Even though having any STI in the last 12 months was significantly associated with being tested for HIV among women, only 3.5% of women and 0.5% of men in our sample reported having any STI in the last 12 months. Since data obtained in the survey were self-reported, the findings could be biased.

In some studies, having two or more sexual partners in the past year is associated with knowledge of HIV status [13, 32]. However, our study did not find this relationship. Instead, among both men and women not knowing the lifetime number of partners was found to be associated with HIV testing. This fact was more significant among women. Sometimes not knowing the number of partners is an indication of having many partners.

A comprehensive knowledge about HIV has been found to be a predictor of HIV testing [7, 8, 13]. Our study shows that men are slightly more knowledgeable about HIV compared with women, including awareness of mother-to-child transmission (MTCT). However, knowledge about MTCT was not found to be significantly associated with HIV testing while, curiously, knowledge of drugs to prevent MTCT was associated with testing.

Our study has some limitations. Causal inferences are limited by the cross-sectional and observational design of the study. Another limitation is the splitting dataset between men and women which might impact on estimates reliability. Also, key high-risk populations are not identified. No information was collected that would identify survey respondents as members of population groups such as sex workers and men having sex with men. These populations are known to be reluctant to report HIV testing [35–37]. This is related to socio-cultural constraints that make these people hide their status fearing rejection and criminalization. HIV infection is often thought to be the result of personal sexual irresponsibility [38] and so is still looked upon as a punishment [39].

New approaches in HIV testing and counselling should be assessed. For Land and al., future diagnostics should incorporate advances in digital technology and mobile health to give REASSURED diagnostic systems [40]. One of these new approaches is HIV self-testing (HIVST), and has been proposed to reach people who are not accessing existing HIV testing services (HTS) such as men, young people and key population groups. Some studies provide more answers. A recent meta-analysis showed HIV self-testing (HIVST) doubled uptake of testing among men [36]. Some studies in Kenya pointed that, HST is feasible in a community level and accepted by the public [41, 42]. So, HIVST can increase uptake and frequency of HIV testing. However, concerns remain about the sensitivity of the test, the lack of council and the need of confirmation after a positive HIV self-test. Despite these concerns, WHO supports the introduction of HIV self-testing as an additional approach to HIV testing services [43]. Nonetheless, our study is important in that it uses nationally representative population survey data. Moreover, it is the first major study on the subject in Senegal.

## Conclusion

This study provides evidence that the HIV testing rate is low in Senegal and that differences in testing persist between men and women. The first UNAIDS “90” target for 2020 has not yet been reached. The main barriers to accessing HIV testing facilities concern problems in reaching men and youth, lack of education and information, low socioeconomic status, rural residence, and poor access to communication.

These findings have implications for policy and programs. Given the low uptake of HIV testing among men and youth, innovative strategies to reach the youngest group, teenagers, must be implemented by scaling up the task with community involvement and access to self-testing tools. Another strategy is scaling up access to HIV testing outside the capital city, especially among women. Furthermore, to address the gaps in knowledge about HIV, communication about HIV and its treatment should be reinforced and women empowered through providing more education and promoting income-generating activities. Also, more integration between HIV and other reproductive health programs must be realized.

## Data Availability

The datasets analyzed during the current study are publicly available upon request at https://dhsprogram.com/data/available-datasets.cfm.

## Abbreviations

ANC: Antenatal care
AOR: Adjusted odds ratio
CI: Confidence intervals
DHS: Demographic Health Survey
HIV/AIDS: Human immunodeficiency Virus/Acquired Immunodeficiency
HIVST: HIV self-testing
MTCT: Mother to child transmission
STI: Sexually transmitted infections
UNAIDS: United Nations Programme on HIV and AIDS
WHO: World Health Organization
